# Supplementary material for: Denoising strategies of functional connectivity MRI data in lesional and non-lesional brain diseases
Source: Imaging Neurosci (Camb). 2025 Oct 31;3:IMAG.a.968. doi: 10.1162/IMAG.a.968 (PMC12580809; doi:10.1162/IMAG.a.968)
Supplement: Supplementary Material [file IMAG.a.968_supp.pdf]

Supplements

Figure 1. Segmentation into White Matter, Grey Matter and Cerebrospinal Fluid

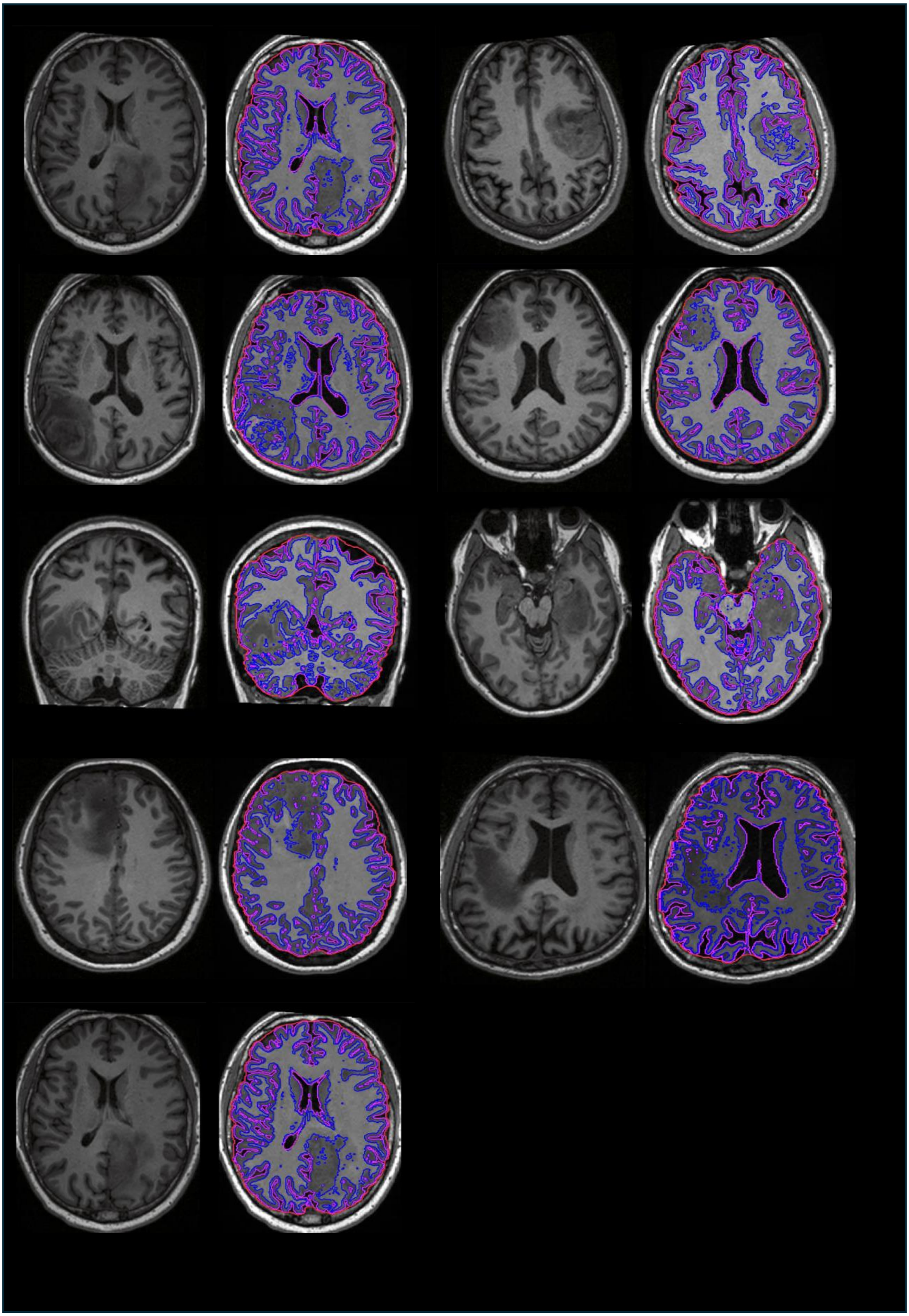

This figure shows the segmentation of T1-weighted images into white, grey matter and cerebrospinal fluid. The blue outlines delineate white matter regions, while the areas between the blue and pink lines represent grey matter.

Table 1. Glioma and Mengioma Characteristics

	**Glioma**	**Meningioma**
**WHO grade**		
Grade I	0	26
Grade II	13	3
Grade III	6	0
Grade IV	15	0
**IDH1/2 mutation status**		
Wildtype	20	
Mutated	14	
**Tumor location**		
Frontal	5	12
Temporal	6	9
Parietal	8	3
Occipital	5	2
Multiple lobes	10	3

This table shows the anatomical location and characteristics of the lesional data.
